# Factors Associated with Smoking Cessation in Patients with Coronary Artery Diseases According to Sex: Cohort of Smoking Cessation Services Data from France

**DOI:** 10.1016/j.cjco.2025.02.002

**Published:** 2025-02-10

**Authors:** Ingrid Allagbé, Marianne Zeller, Daniel Thomas, Guillaume Airagnes, Frédéric Limosin, Abdelali Boussadi, Frédéric Chagué, Anne-Laurence Le Faou

**Affiliations:** a Physiopathology and Epidemiology Cerebro-Cardiovascular, PEC2, EA 7460 UFR Health Sciences, University of Burgundy and Franche Comté, Dijon, France; b Scientific Interest Group of the French Network of Excellence for Research on Tobacco, Nicotine and Related Products (GIS REfer Tab), Paris, France; c Cardiology Department, Dijon University Hospital, Dijon, France; d Université Paris-Sorbonne, AP-HP, Institut de Cardiologie, Hôpital Pitié-Salpêtrière, Paris, France; e University Paris Cité, AP-HP. Center, Outpatient Addictology Center, Paris, France; f DMU Psychiatry and Addictology, AP-HP.Centre-University of Paris, Paris, France; g Département de Santé Publique et Informatique Médicale, Hôpital Européen Georges Pompidou, AP-HP. Centre—Université de Paris, Paris, France; h Fédération Hospitalo-Universitaire Network of Research in Substance Use Disorder, Assistance Publique-Hôpitaux de Paris, Paris, France

## Abstract

**Background:**

In smokers with coronary artery diseases (CADs), smoking cessation (SC) is a major prevention goal. From the French national database of SC services (SCSs), CDTnet, we aimed to describe the social, medical, and smoking characteristics of smokers with CAD, as well as factors associated with their SC, according to sex.

**Methods:**

A retrospective study was conducted of smokers with CAD included in the CDTnet from January 2001 to December 2018. Endpoints were abstinence and reduction of daily cigarette consumption. Abstinence was defined as SC maintained for ≥ 28 consecutive days, confirmed by a carbon monoxide measure in exhaled breath testing < 10 parties per million, and reduction was defined as at least a halving of consumption compared to consumption at the time of the first consultation. Sex stratification was performed.

**Results:**

Among 4532 smokers included, 21% were women, and their mean age was 55 years in both sexes. Nearly half smoked ≥ 20 cigarettes daily, and most (80%) received nicotine replacement therapy. The 28-day abstinence rate (54%) and reduction rate (24%) were similar in both sexes. Factors positively associated with SC in women were having made ≥ 1 previous quit attempt, and dual use of conventional and electronic cigarettes at the time of the first consultation. In men, being employed, being overweight or obese, being confident in quitting, and being prescribed nicotine replacement therapy at the first consultation were factors associated with success. Other cardiovascular and respiratory diseases were associated negatively with SC in both sexes.

**Conclusions:**

Abstinence rates were similar for both sexes, with different factors associated with quit attempt results, according to sex, highlighting the need for tailored interventions that address the specific needs of men and women who intend to quit.

## Graphical abstract

**Figure:**
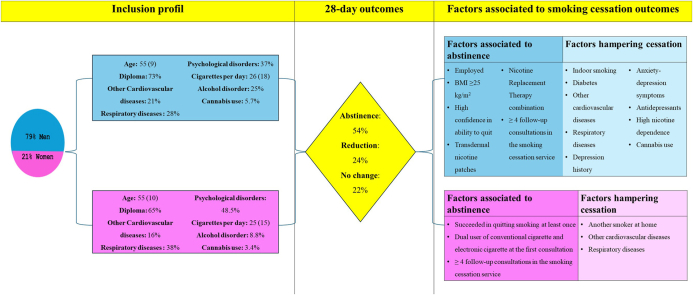

## Résumé

**Contexte:**

Chez les fumeurs atteints de maladies coronariennes (MC), le sevrage tabagique (ST) est un objectif de prévention majeur. À partir de la base de données nationale française de Consultations de Dépendance Tabagique, CDTnet, nous avons cherché à décrire les caractéristiques sociales, médicales et tabagiques des fumeurs atteints de MC, ainsi que les facteurs associés à leur ST, en fonction du sexe.

**Méthodologie:**

Une étude rétrospective a été menée sur les fumeurs atteints de MC inclus dans CDTnet, de janvier 2001 à décembre 2018. Les critères d'évaluation étaient l'abstinence et la réduction de la consommation quotidienne de cigarettes. L'abstinence était définie comme un ST maintenu pendant ≥ 28 jours consécutifs, confirmé par une mesure de monoxyde de carbone dans l'air expiré < 10 particules par million, et la réduction était définie comme une diminution d'au moins la moitié de la consommation de cigarettes par rapport à la consommation déclarée au moment de la première consultation. Une stratification par sexe a été réalisée.

**Résultats:**

Parmi les 4 532 fumeurs inclus, 21 % étaient des femmes. L'âge moyen était de 55 ans pour les deux sexes. Près de la moitié fumait ≥ 20 cigarettes par jour, et la plupart (80 %) ont reçu une prescription de substituts nicotiniques. Le taux d'abstinence maintenue 28 jours (54 %) et le taux de réduction (24 %) étaient similaires dans les deux sexes. Les facteurs positivement associés au ST chez les femmes étaient le fait d'avoir fait ≥ 1 tentative d'arrêt antérieure, et la double utilisation de cigarettes conventionnelles et électroniques au moment de la première consultation. Chez les hommes, le fait d'avoir un emploi, d'être en surpoids ou obèse, d'être confiant dans l'arrêt du tabac et de se voir prescrire un traitement de substitution nicotinique lors de la première consultation étaient des facteurs associés à la réussite du ST. Les autres maladies cardiovasculaires et respiratoires déclarées étaient associées négativement au ST dans les deux sexes.

**Conclusions:**

Les taux d'abstinence étaient similaires pour les deux sexes, avec différents facteurs associés aux résultats des tentatives d'arrêt selon le sexe, soulignant la nécessité d'interventions adaptées qui répondent aux besoins spécifiques des hommes et des femmes qui ont l'intention d'arrêter de fumer.

Despite sustained declines in the incidence of mortality from cardiovascular diseases (CVDs) in many countries, CVDs, including coronary artery diseases (CADs), have remained the most common cause of death, worldwide^1^ and across Europe.^2^ Both active and passive smoking are major risk factors for CAD, including acute myocardial infarction (MI) and angina.^3^ Smoking significantly elevates oxidative stress levels, promotes vascular inflammation, induces vasoconstriction, and enhances platelet reactivity.^4^^,^^5^ These factors contribute to vascular endothelial dysfunction, which can impair blood vessel function and increase the risk of CAD.^4^^,^^5^

In fact, smokers are 2 to 4 times more likely to develop CAD, as compared with nonsmokers; this smoking-related coronary risk is highest in smokers aged 35-54 years, and decreases significantly at ages thereafter.^6^ Furthermore, the smoking-related excess coronary risk is dose-dependent, with each gram of tobacco smoked daily increasing the risk of first MI by 2%-3%.^7^ Finally, this excess risk is more marked in women than it is in men. In fact, female smokers have 1.25 times the risk of CAD, compared with that for male smokers.^8^

In smokers with CAD, smoking cessation (SC) is one of the main factors that reduce the risk of recurrence or death, regardless of both the severity of the CAD and the duration of tobacco exposure.^9^ In fact, according to a study involving smokers who survived their first MI and were followed for 5 years (or until death, if it occurred sooner), the age- and sex-adjusted mortality rates for the combined group of men and women indicated that individuals who quit smoking after their MI experienced a mortality rate that was 55% of the rate for those who continued to smoke.^10^ Moreover, among all secondary prevention strategies, including pharmacologic treatments, SC carries the best ratios of both cost to effectiveness and cost to benefit.^11^ However, despite this high degree of public health impact, only very limited data are available on the specifics and determinants of successful SC in CAD patients.

Thus, based on the French nationwide database of SC services (SCS),^12^ we aimed to describe the social, medical, and smoking characteristics of smokers with CAD, and to identify the factors associated with SC, with a comparative focus on men vs women.

## Materials and Methods

This study was observational and used anonymized data recorded in the French SCS national database—"Consultations de dépendance tabagique" (CDT)net.^12^ For this study, 72 SCSs contributed continuously to CDTnet in the study period; most (96.3%) were located in public hospitals. Additionally, a small proportion of SCSs were located in addictology centres (1.3%), prison health services (1.2%), private hospitals (0.8%), general practitioners’ practices (0.3%), and medical dispensaries (0.2%).

During their first consultation in an SCS, since 2000, smokers are invited to fill in a standardized paper questionnaire, developed at the nationwide level by a working group at the initiative of Santé Publique France. The information provided by the smoker is checked by the staff of the SCS and recorded in the CDTnet database. Then, the smokers included receive follow-up assessment during their attempt to quit. The follow-up data and the patient’s smoking status are systematically recorded in CDTnet at each consultation.

### Usual care

Concerning SCS, smokers are offered behavioural support and pharmacologic treatment with 45-60 minutes dedicated for the first consultation, and 30 minutes for the follow-up consultations. The support includes the following behaviourchange techniques, per the Michie et al. taxonomy^13^: facilitation of action planning and goal setting; review of outcome goals; pharmacologic support if the smoker accepts it; information about the health consequences of smoking and SC; and biofeedback (carbon monoxide [CO] monitoring).^14^

### Inclusion criteria

We included current smokers registered in CDTnet from January 1, 2001 to December 31, 2018, aged ≥ 18 years at the time of the first consultation, who reported a history of MI or angina pectoris and had completed at least 28 days of follow-up in an SCS.^15^ Being a current smoker was defined as reporting smoking combustible tobacco (cigarillo; rolled cigarette; manufactured cigarette) either “every day” or “some days” at the time of the first consultation.^16^ Participants who were non-smokers at inclusion, as well as minors and pregnant women, were not included in the study.

### Variable definitions

The following sociodemographic profile variables were collected: sex^1^; age divided into classes (18-34 years, 35-64 years, ≥ 65 years); professional activity (in 2 classes—active vs non-active—with active defined as being employed at the time of the first consultation); and diploma holder (no diploma, or vocational school diploma (Brevet d’Etudes Professionnelles [BEP] and Certificat d’aptitude professionnelle [CAP]—which correspond to a technical vocational high school diploma received after 2 years of study), high school diploma holder, or higher education completion (≥ 2 years of study after high school). For medical data, smokers indicated the existence of a cardiovascular risk factor (ie, hypertension, diabetes, hypercholesterolemia or a body mass index [BMI] ≥ 25 kg/m^2^),^17^ and a history of CVD other than MI and angina that is, stroke and peripheral arterial disease—as well as psychological data, including history of depression, the presence of anxiety and/or depression symptoms, and intake of psychotropic medications. The Hospital Anxiety and Depression Scale (HAD) was used to screen patients for anxiety and/or depressive disorders. A HAD anxiety score ≥ 11 suggests an anxiety state, and a HAD depression score ≥ 8 suggests a depressive state.^18^ Other pathologies recorded were respiratory diseases (chronic obstructive pulmonary disease, chronic bronchitis, asthma) and smoking-related cancers recorded in CDTnet (ie, lung cancer, oropharyngeal cancer, bladder cancer).

Regarding smoking behaviours, we collected information on the following factors: the origin of the consultation (personal initiative and/or request from family and friends; referral by hospital and/or primary care health professional); number of previous quit attempts of > 7 days (0 attempts; ≥ 1 attempt), number of cigarettes smoked per day (≤ 20 cigarettes; > 20 cigarettes [termed “high cigarette consumption”]). A cigarillo was considered to be equivalent to 2 manufactured cigarettes, and a rolled cigarette was considered to be equivalent to 2 manufactured cigarettes.^19^

Confidence in the ability to quit smoking was measured by a visual analog scale, scored from 0 to 10—a high level of confidence was defined by a score ≥ 6. Nicotine dependence was measured by the Heaviness of Smoking Index, with a cutoff ≥ 4 indicating a high level of nicotine dependence^20^; Smoking abstinence was validated by a CO measure of < 10 parties per million (ppm) in exhaled air at each consultation. The existence of co-addictions (ie, alcohol use disorder, cannabis use, and intake of opioid substitution treatment) was recorded. Alcohol use disorder was defined as a consumption of ≥ 2 drinks per day, regardless of gender,^21^ and data on cannabis use in the past 12 months were collected. Since October 2015, a dual use of conventional cigarettes and electronic cigarettes with nicotine-containing or non-nicotine-containing liquid was registered at the first consultation. Validated SC treatments received at the first consultation were collected and then classified into 8 classes, as follows: psychosocial support (PS) with cognitive behavioural techniques without any SC pharmacologic treatment; transdermal nicotine patch + PS; oral nicotine substitute + PS; nicotine replacement therapy (NRT) combination + PS; varenicline + PS; varenicline + NRT + PS; bupropion + PS; and bupropion + NRT + PS. Finally, the number of follow-up consultations was recorded.

### Endpoints

The primary outcome was 28-day smoking abstinence, defined as self-reported SC of ≥ 28 consecutive days during follow-up consultations, confirmed by measurement of exhaled CO of < 10 ppm at each follow-up consultation.^15^ Finally, we also use the criteria for English SCS effectiveness evaluation, which show that 1-month abstinence predicts 1-year abstinence.^22^ A secondary outcome was defined as a reduction in smoking, defined as halving cigarette consumption since the first consultation.^23^

### Management of missing data

For the CO variable, which contained 11% missing data, we performed multiple imputation.

### Statistical methods

The distribution of the data was evaluated using the Agostino test. A Student's *t*-test or a Mann–Whitney U test was used for continuous variables, and the raw data were described using their mean and standard deviation. A χ^2^test was used for categorical variables, with results presented using numbers and percentages. In a second step, we performed unconditional univariate logistic regression by modelling the binary variable for abstinence (yes or no). The “yes” group corresponded to smokers who met the abstinence criteria defined above. The “no” group included smokers who had reduced their cigarette consumption by 50%—as compared to their cigarette consumption at the first consultation—and those who had continued to smoke without reduction. In addition, we performed a CAR ANOVA model to explore the interaction of abstinence with sex. Furthermore, we performed a multivariate stepwise logistic regression. For the regressions, “abstinence” (yes or no) was studied with adjustment for sociodemographic and clinical characteristics, characteristics related to smoking behaviour, and SC treatment received at the first consultation. The different risk outcomes are presented as odds ratios (ORs) with their 95% confidence intervals (CIs). The multicollinearity of variables was tested by measuring variance inflation factors (VIF). All variables had a VIF close to 1, allowing rejection of the multicollinearity hypothesis. Our results were described by sex. Finally, we performed a subgroup analysis for those who had dual use of conventional cigarettes and electronic cigarettes at the first consultation. The analyses were performed with R software, version 4.2.1, R Foundation, Vienna, Austria.

### Ethics

All patients included in the database had given their consent before the collection and recording of their data in the CDTnet database. In addition, this database is authorized by the French data protection agency (Commission Nationale de l'Informatique et des Libertés; CNIL; authorization #739406). Additional information on the CDTnet database is available in a previous publication.^24^

## Results

### Population

**Figure 1:**
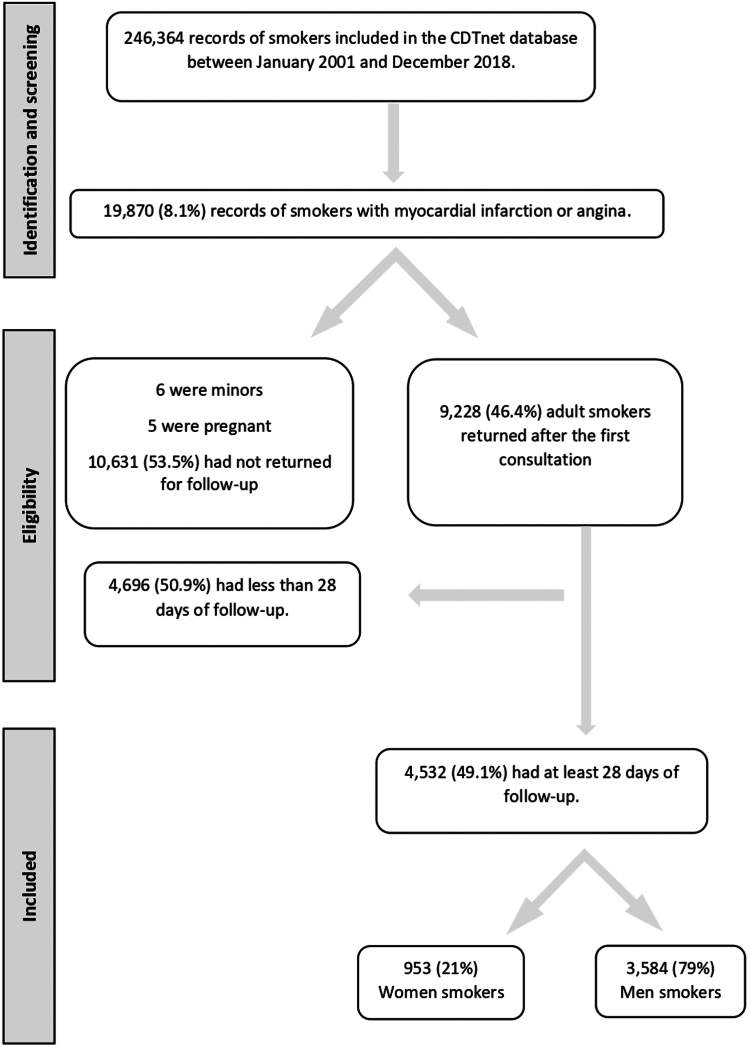
Flowchart of smokers included in the study. CDTnet, Consultations de dépendance tabagique (French national database of smoking cessation services).

During the inclusion period, 19,870 initial consultations of smokers with MI or angina were recorded in the CDTnet database. Among these smokers, < 1% (N = 6) were minor smokers, and more than a half (53.5 %) did not return to SCSs for follow-up. This dropout rate was expected, as the literature indicates that only half of smokers return for a follow-up visit at a SCS.^25^ Among the remaining smokers, 4696 (50.9 %) were followed-up for < 28 days. Consequently, among smokers suffering from MI or angina, as recorded in the CDTnet database January 1, 2001 to December 31, 2018, 22.8% (N = 4532) met the inclusion criteria. Thus, our study included 4532 smokers, of whom 21% were women (Fig. 1). The mean follow-up duration was 291.6 days, and the median duration was 106 days.

### Sample description

Baseline characteristics of the patients are presented in Table 1. The mean age of the study population was 55 years (± 9), and it was similar for both sexes. Two-thirds of the patients had consulted an SCS after contact with a hospital and/or health professional, and almost half of them (45%) smoked > 20 cigarettes per day. Compared to men, women were less likely to have a diploma (65% vs 73%; *P* < 0.001), to be employed (43% vs 48%; *P* = 0.010), and to suffer from a CVD other than MI and angina (16% vs 21%; *P* < 0.001). Conversely, women were more likely to have respiratory diseases (38% vs 28%; *P* < 0.001), a history of depression (40% vs 23%; *P* < 0. 001), and anxiety and/or depression symptoms (57% vs 41%; *P* < 0.001). Regarding co-addictions, women were markedly less likely to have an alcohol use disorder (9% vs 25%; *P* < 0.001) and also to have used cannabis in the past year (3.4% vs 5.7%; *P* < 0.004). Finally, women smoked fewer cigarettes per day, compared to men, but they had a higher level of nicotine dependence and were less confident about quitting.

**Table 1:** Demographic, clinical, and smoking-behaviour profile of the study population

Characteristics	Women, N = 948	Men, N = 3584	Overall, N = 4532	*P*
**Age, y, mean (SD)**	55 (10)	55 (9)	55 (9)	0.095
**Age classes, y**				0.8
18–34	16 (1.7)	51 (1.4)	67 (1.5)	
35–64	783 (83)	2984 (83)	3767 (83)	
≥ 65	149 (16)	549 (15)	698 (15)	
**Contraceptive pill**	35 (3.7)		35 (0.8)	< 0.001
**Diploma**	613 (65)	2631 (73)	3244 (72)	< 0.001
**Employed**	407 (43)	1706 (48)	2113 (47)	0.010
**Personal initiative or encouraged by entourage**	243 (26)	807 (23)	1050 (23)	0.043
**Smokes at home**	87 (9.2)	218 (6.1)	305 (6.7)	< 0.001
**Other smoker at home**	49 (5.2)	116 (3.2)	165 (3.6)	0.005
**Cardiovascular risk factors**				
BMI ≥ 25 kg/m^2^	461 (49)	2,079 (58)	2,540 (56)	< 0.001
Arterial hypertension	359 (38)	1,228 (34)	1,587 (35)	0.038
Diabetes	148 (16)	628 (18)	776 (17)	0.2
Hypercholesterolemia	390 (41)	1,657 (46)	2,047 (45)	0.005
**Other cardiovascular diseases**	150 (16)	755 (21)	905 (20)	< 0.001
**Respiratory diseases**	362 (38)	1,002 (28)	1,364 (30)	< 0.001
**Psychological disorders**				
Depression history	379 (40)	832 (23)	1,211 (27)	< 0.001
Anxiety and/or depression symptoms∗	544 (57)	1,471 (41)	2,015 (44)	< 0.001
**Psychotropic medications**				
Anxiolytics	310 (33)	770 (21)	1,080 (24)	< 0.001
Antidepressants	268 (28)	618 (17)	886 (20)	< 0.001
**Smoking-related cancers**	45 (4.7)	142 (4.0)	187 (4.1)	0.3
**Prior attempt to quit**	684 (72)	2,601 (73)	3,285 (72)	0.8
**Cigarettes per day, mean (SD)**	25 (15)	26 (18)	26 (17)	0.085
**High level of cigarette consumption**†	395 (42)	1,657 (46)	2,052 (45)	0.012
**High level of nicotine dependence**‡	613 (65)	2,167 (60)	2,780 (61)	0.018
**Confidence in ability to quit**§**, mean (SD)**	4 (3)	5 (3)	4 (3)	<0.001
**AverageexpiredCOlevelat the first consultation(parts per million), mean(SD)**	14 (14)	15 (14)	14 (14)	0.2
**Co-addictions**				
Alcohol use disorder	83 (8.8)	885 (25)	968 (21)	<0.001
Cannabis use	32 (3.4)	206 (5.7)	238 (5.3)	0.004
**Opioid substitution treatment**	9 (0.9)	31 (0.9)	40 (0.9)	0.8
**Use of electronic cigarettes at the first consultation (dual users)**	32 (3.4)	84 (2.3)	116 (2.6)	0.074
**Treatment prescribed at the first consultation**				0.2
PS No pharmacotherapy	139 (15)	444 (12)	583 (13)	
Transdermal nicotine patches + PS	182 (19)	656 (18)	838 (18)	
Oral nicotine substitutes + PS	129 (14)	481 (13)	610 (13)	
NRT Combination + PS	444 (47)	1,824 (51)	2,268 (50)	
Varenicline + PS	39 (4.1)	138 (3.9)	177 (3.9)	
Varenicline + NRT + PS	13 (1.4)	35 (1.0)	48 (1.1)	
Bupropion + PS	1 (0.1)	3 (<0.1)	4 (<0.1)	
Bupropion + NRT + PS	1 (0.1)	3 (<0.1)	4 (<0.1)	
**Number of follow-up consultations**				
Mean (SD)	5 (6)	4 (6)	5 (6)	0.2
1–3	517 (55)	2057 (57)	2574 (57)	0.3
4–6	239 (25)	828 (23)	1067 (24)	
≥ 7	192 (20)	699 (20)	891 (20)	
**Smoking status**				0.6
Abstinence	504 (53)	1932 (54)	2436 (54)	
Reduction	224 (24)	872 (24)	1096 (24)	
No change in consumption	220 (23)	780 (22)	1000 (22)	

Values are n (%), unless otherwise indicated.

BMI, body mass index; NRT, nicotine replacement therapy; PS, psychosocial support; SD, standard deviation.

∗ Anxiety and/or depression symptoms as measured by the Hospital Anxiety Depression Scale.

† >20 cigarettes smoked per day.

‡ Heaviness of Smoking Index ≥4.

§ Measured by a visual analog scale scored from 0 to 10.

### Outcomes at the end of follow-up: univariate analyses

**Figure 2:**
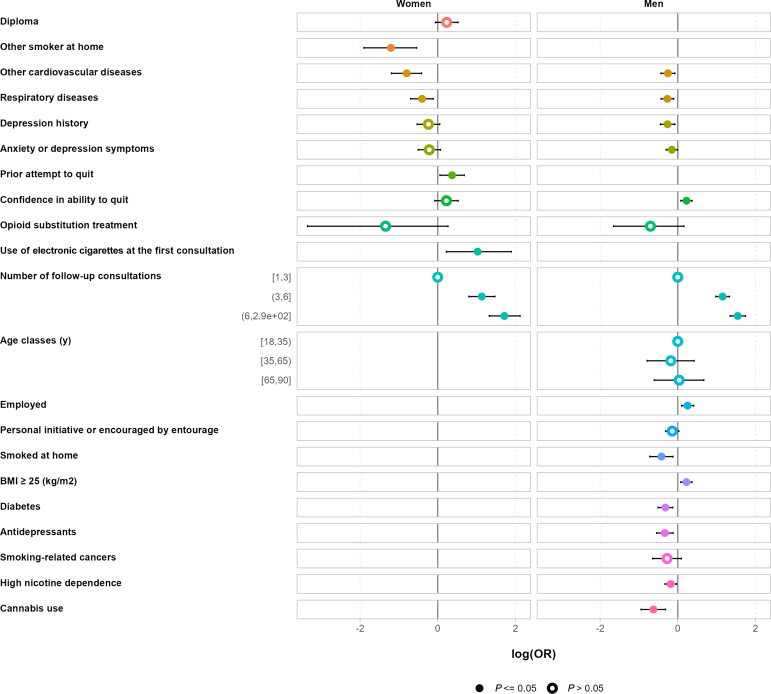
Factors associated with smoking cessation in women and men; results of the stepwise multivariate regression analysis. BMI, body mass index; OR, odds ratio.

Table 2 and Supplemental Table S1 show the characteristics of the study population according to SC achievement and sex. At the 28-day follow-up evaluation, many participants were found to have improved their smoking behaviour, with an abstinence rate of 54% (53% women vs 54% men) of our study population, with no significant difference between men and women (*P* = 0.6). The best abstinence rates were observed in smokers of both sexes who had a diploma (55%), were employed (57%), presented with a BMI ≥ 25 (55%), who already had made ≥ 1 quit attempt and those had been prescribed transdermal nicotine patches, an NRT combination, or varenicline at the first consultation (Table 2). In contrast, the following subgroups of smokers obtained low SC rates: smokers with diabetes (48%); those presenting with CVDs other than MI and angina (46%); smokers suffering from respiratory diseases (48%); smokers presenting with a depression history (47%) or intake of psychotropic medication (48%); smokers who had used cannabis in the previous year (38%); and those receiving opioid substitution treatment (25%; Table 2). The mean exhaled CO level measured at the first consultation was 13 ppm for the abstainers, 17 ppm for those who had reduced their consumption, and 15 ppm for the others (Table 2; Fig. 2). The proportion of smokers who reduced their tobacco consumption was 24% in both sexes (*P* = 0.6). Men and women smokers receiving opioid substitution treatment were more likely to try to reduce, rather than quit, their tobacco consumption (35% vs 25%, *P* < 0.001; Table 2). Compared to men, women who lived with another smoker more often reduced vs quit their tobacco consumption (women [abstinence, 35% vs reduction, 53%]; men [abstinence, 45% vs reduction, 41%]) (Supplemental Table S1).

**Table 2:** Outcomes at the end of the follow-up period

Characteristics	Abstinence, N = 2469^1^	Cigarette consumption reduction, N = 1095^1^	No change in cigarette consumption, N = 968^1^	*P*^2^
**Sex**				0.6
Female	504 (53)	224 (24)	220 (23)	
Male	1932 (54)	872 (24)	780 (22)	
**Age,y,mean (SD)**	55 (9)	56 (9)	54 (9)	< 0.001
**Age classes, y**				0.004
18–34	38 (57)	10 (15)	19 (28)	
35–64	2007 (53)	899 (24)	861 (23)	
≥ 65	391 (56)	187 (27)	120 (17)	
**Contraceptive pill**	23 (66)	5 (14)	7 (20)	0.3
**Diploma**	1790 (55)	813 (25)	641 (20)	< 0.001
**Employed**	1211 (57)	428 (20)	474 (22)	< 0.001
**Personal initiative or encouraged by entourage**	559 (53)	264 (25)	227 (22)	0.7
**Smokes at home**	131 (43)	116 (38)	58 (19)	< 0.001
**Other smoker at home**	69 (42)	74 (45)	22 (13)	< 0.001
**Cardiovascular risk factors**				
BMI ≥ 25 kg/m^2^	1,403 (55)	612 (24)	525 (21)	0.024
Arterial hypertension	811 (51)	409 (26)	367 (23)	0.031
Diabetes	369 (48)	231 (30)	176 (23)	< 0.001
Hypercholesterolemia	1096 (54)	515 (25)	436 (21)	0.3
**Cardiovascular diseases**	416 (46)	271 (30)	218 (24)	< 0.001
**Respiratory diseases**	653 (48)	371 (27)	340 (25)	< 0.001
**Psychological disorders**				
Depression history	567 (47)	331 (27)	313 (26)	< 0.001
Anxiety and/or depression symptoms∗	1019 (51)	536 (27)	460 (23)	< 0.001
**Psychotropic medications**				
Anxiolytics	525 (49)	303 (28)	252 (23)	< 0.001
Antidepressants	405 (46)	265 (30)	216 (24)	< 0.001
**Smoking-related cancers**	89 (48)	56 (30)	42 (22)	0.13
**Prior attempt to quit**	1786 (54)	788 (24)	711 (22)	0.4
**Cigarettes per day, mean (SD)**	25 (16)	27 (20)	26 (17)	0.042
**High level of cigarette consumption**†	1356 (55)	593 (24)	531 (21)	0.3
**High level of nicotine dependence**‡	1451 (52)	688 (25)	641 (23)	0.055
**High level of confidence in ability to quit**§	965 (57)	419 (25)	298 (18)	< 0.001
**AverageexpiredCOlevelat the first consultation(parts per million), mean (SD)**	13 (14)	17 (14)	15 (14)	< 0.001
**Co-addictions**				
Alcohol use disorder	508 (52)	250 (26)	210 (22)	0.4
Cannabis use	90 (38)	78 (33)	70 (29)	< 0.001
**Opioid substitution treatment**	10 (25)	14 (35)	16 (40)	< 0.001
**Use of electronic cigarettes at the first consultation (dual users)**	58 (50)	36 (31)	22 (19)	0.2
**Treatment prescribed at the first consultation**				< 0.001
PS No pharmacotherapy	283 (49)	128 (22)	172 (30)	
Transdermal nicotine patches + PS	478 (57)	210 (25)	150 (18)	
Oral nicotine substitutes + PS	238 (39)	187 (31)	185 (30)	
NRT combination + PS	1307 (58)	511 (23)	450 (20)	
Varenicline + PS	103 (58)	40 (23)	34 (19)	
Varenicline + NRT + PS	21 (44)	20 (42)	7 (15)	
Bupropion + PS	2 (50)	0 (0)	2 (50)	
Bupropion + NRT + PS	4 (100)	0 (0)	0 (0)	
**Number of follow-up consultations**				
Mean (SD)	6 (6)	4 (5)	2 (2)	< 0.001
1–3	1056 (41)	669 (26)	849 (33)	
4–6	715 (67)	249 (23)	103 (9.7)	
≥ 7	665 (75)	178 (20)	48 (5.4)	

Values are n (%), unless otherwise indicated.

BMI, body mass index; NRT, nicotine replacement therapy; PS, psychosocial support; SD, standard deviation.

∗ Anxiety and/or depression symptoms as measured by the Hospital Anxiety Depression Scale.

† >20 cigarettes smoked per day.

‡ Heaviness of Smoking Index ≥4.

§ Measured by a visual analog scale scored from 0 to 10.

### Results of sex-interaction analysis

Supplemental Table S2 shows 4 variables that were found to have an interaction with sex, as follows: living with another smoker (likelihood ratio [LR] χ^2^ =3.92; *P* = 0.048); presenting other cardiovascular diseases (LR χ^2^ = 4.40; *P* = 0.036); declaring a prior attempt to quit (LR χ^2^ = 6.78, *P* = 0.009), using an electronic cigarette at the first consultation (LR χ^2^ = 8.10, *P* = 0.004; Supplemental Table S2) Supplemental Figure S1 corresponds to the representation of the 4 variables that show an interaction with sex.

### Outcomes at the end of follow-up: regression models

Results of unconditional univariate logistic regression are presented in Supplemental Table S3. Table 3 and Figure 2 present the results of the multivariate stepwise logistic regression model analysis, according to sex. Among women, the factors associated with abstinence were having made ≥ 1 previous quit attempt (OR = 1.56; 95% CI [1.14-2.15] and being dual users of conventional cigarettes and electronic cigarettes at the first consultation (OR = 2.75; 955 CI [1.22-6.61]). The only factor associated with persistent smoking in women was the presence of another smoker at home (OR = 0.28; 955 CI [0.14-0.55]).

**Table 3:** Factors associated with smoking cessation in women and men; results of the stepwise multivariate regression analysis

Characteristics	Women		Men	
	OR^10^	95% CI^11^	OR adjusted^12^	95% CI^13^
**Age classes, y**				
18–34				
35–64				
≥ 65				
**Contraceptive pill**				
**Diploma**				
**Employed**			1.22	1.05, 1.42
**Personal initiative or encouraged by entourage**			0.88	0.74, 1.04
**Smoked at home**			0.61	0.45, 0.83
**Other smoker at home**	0.28	0.14–0.55		
**Cardiovascular risk factors**				
BMI ≥ 25 kg/m^2^			1.24	1.07, 1.44
Arterial hypertension				
Diabetes			0.73	0.60, 0.88
Hypercholesterolemia				
**Other cardiovascular diseases**	0.45	0.30–0.66	0.78	0.65, 0.93
**Respiratory diseases**	0.65	0.48–0.88	0.78	0.66, 0.92
**Psychological disorders**				
Depression history	0.78	0.58–1.05	0.78	0.64, 0.94
Anxiety or depression symptoms∗	0.79	0.59–1.06	0.86	0.74, 1.01
**Psychotropic medications**				
Anxiolytics			0.86	0.71, 1.05
Antidepressants			0.77	0.62, 0.97
**Smoking-related cancers**				
**Prior attempt to quit**	1.56	1.14–2.15		
**High cigarette consumption**†				
**High nicotine dependence**‡			0.78	0.67, 0.91
**High confidence in ability to quit**§	1.27	0.94–1.73	1.21	1.04, 1.40
**Co-addictions**				
Alcohol use disorder				
Cannabis use			0.52	0.38, 0.71
**Opioid substitution treatment**	0.21	0.03–1.17	0.51	0.19, 1.21
**Use of electronic cigarettes at the first consultation**	2.75	1.22–6.61		
**Treatment prescribed at the first consultation**				
PS No pharmacotherapy	—	—	—	—
Transdermal nicotine patches + PS	0.88	0.54–1.43	1.63	1.26, 2.12
Oral nicotine substitutes + PS	0.47	0.28–0.80	0.85	0.64, 1.12
NRT Combination + PS	1.21	0.80–1.84	1.58	1.26, 1.98
Varenicline + PS	2.07	0.93–4.88	1.28	0.85, 1.93
Varenicline + NRT + PS	0.50	0.13–1.80	0.79	0.38, 1.67
Bupropion + PS	661,145	0.00 – NA	0.29	0.01, 3.24
Bupropion + NRT + PS	859,046	0.00 – NA	188,560	0.00, NA
**Number of follow-up consultations**				
1–3	—	—	—	—
4–6	3.01	2.15–4.25	3.07	2.58, 3.67
≥ 7	5.54	3.74–8.35	4.75	3.90, 5.83

CI, confidence interval; NA, not applicable; NRT, nicotine replacement therapy; OR, odds ratio; PS, psychosocial support.

∗ Anxiety and/or depression symptoms as measured by the Hospital Anxiety Depression Scale.

† >20 cigarettes smoked per day.

‡ Heaviness of Smoking Index ≥4.

§ Measured by a visual analog scale scored from 0 to 10.

Among men, the factors associated with abstinence were as follows: being employed (OR = 1.22; 95% CI [1.05-1.42]), being overweight or obese (OR = 1.24; 95% CI [1.07-1.44]), having confidence in their ability to quit (OR = 1.21; 955 CI [1.04-1.40]), and having received a transdermal nicotine patch or an NRT combination prescription at the first consultation. Conversely, the factors hampering abstinence in men were as follows: smoking at home (OR = 0.61; 95% CI [0.45-0.83]); suffering from diabetes (OR = 0.73; 95% CI [0.60-0.88]); presenting with a depression history (OR = 0.78; 95% CI [0.64-0.94]); taking antidepressants (OR = 0.77; 95% CI [0.62-0.97]); having a high level of nicotine dependence (OR = 0.78; 95% CI [0.67-0.91]); and having used cannabis during the previous year (OR = 0.52; 95% CI [0.38-0.71]; Table 3; Fig. 2).

Finally, in both sexes, the factor associated positively with abstinence was having made several follow-up consultations in the SCS (≥ 7 follow-up consultations: OR = 5.54; 95% CI [3.74-8.35] in women vs OR = 4.75; 95% CI [3.90-5.83] in men). Factors negatively associated with abstinence in both sexes were as follows: presenting with another CVD (OR = 0.45; 95% CI [0.30 - 0.66] in women vs OR = 0.78; 95% CI [0.65-0.93] in men), as well as suffering from a respiratory disease (OR = 0.65; 95% CI [0.48-0.88] in women vs OR = 0.78; 95% CI [0.66-0.92] in men).

## Discussion

This study investigated the profile and predictors of SC among smokers with MI or angina who sought help to quit at an SCS between 2001 and 2018. The 28-day abstinence rate was 54%, and a reduction in tobacco consumption was obtained in 24% of smokers, without any difference according to sex. Among women, having already succeeded in quitting smoking at least once, and being a dual user of conventional and electronic cigarettes at the first consultation were factors positively associated with abstinence, whereas the presence of another smoker at home was associated negatively with quitting. Conversely, in men, factors associated with abstinence were being employed, having a BMI ≥25 kg/m^2^, being confident in their ability to quit, and benefiting from an NRT prescription, patch, or NRT combination. Factors hampering cessation in men were as follows: indoor smoking, presenting with diabetes, depression history, anxiety and/or depression symptoms, taking antidepressants, having a high level of nicotine dependence, and cannabis use.

Our population included 21% women, which is consistent with CVD epidemiologic data in the general population, as women have lower absolute rates of CAD than men, as observed in France.^6^^,^^26^

The smoking profile of smokers in our study was more severe than that of the smokers in the French general population.^27^ Indeed, smokers consumed an average of 26 (± 17) cigarettes per day, and 61% of the study population had a high level of nicotine dependence, whereas in the general population, adult smokers consume an average of 12.5 cigarettes per day.^27^ These data could be explained by the characteristics of smokers who were referred to an SCS, as they are smokers who have difficulty quitting, given their profile of severe smoking behaviour with a high level of cigarette consumption and nicotine dependence.^28^

In our study, NRT was the only SC medication that was associated positively with SC in men. Our results are consistent with those in the literature suggesting that use of NRT is more effective for SC in men, whereas use of varenicline and bupropion are more effective in women.^29^^,^^30^

Almost three-quarters of our population had made at least 1 previous quit attempt, with no significant difference between men and women. However, this variable was associated positively with SC only in women. Being a dual user of conventional cigarettes and electronic cigarettes at the first consultation was also associated positively with SC in women. This difference between men and women could be explained by the fact that female dual users were more likely than men to seek help in quitting from an SCS, possibly because they could not quit smoking completely using electronic cigarettes alone. Furthermore, SC teams may inform smokers of the cardiovascular risk associated with a dual use of conventional and electronic cigarettes during hospital bedside consultations. Indeed, compared to exclusive smokers, dual users—of both conventional and electronic cigarettes—have been found to have a higher risk of CAD than do exclusive smokers.^31^ Nevertheless, dual use also appears to be associated with an increase in the success of quitting attempts, which could explain the abstinence rate in our data among women who were dual users.^32^

The existence of a depression history was associated negatively with SC in men. Furthermore, men and women in our study might have had different depression backgrounds. One possibility is that men included in our study had a history of recurrent depression and therefore corresponding higher levels of baseline depressive symptoms, compared to those in women.^33^ Finally, men were more numerous than women in our study, which may explain the negative association between depression history and SC, unless this result is the opposite of what was found in the literature.^34^

Compared with the average SC rate in the study, smokers with other comorbidities had a lower SC rate, compared to that in smokers with solely CAD, in both sexes. Despite these low levels of SC in CAD smokers with comorbidities (46%-48%), the validated 28-day abstinence rate was 54% among the study participants. This result is important, as persistent smoking following a CAD diagnosis is a major contributing factor to mortality and major adverse cardiac events, especially among women.^35^

### Limitations and strengths

This study has several limitations. The main bias of this study is that smokers seeking help in quitting from an SCS might be more motivated to quit, creating a potential selection bias. This possibility prevents generalization of our results to the population of smokers with CAD. Another limitation of this study concerns the treatments that may have been prescribed during follow-up consultations. In fact, only data on SC treatment prescribed at the first consultation were collected. Consequently, smokers could have used additional SC treatments as a means to quit smoking during the follow-up period, which could have had an effect on the 28-day SC rate. However, this limitation does not affect the quality of the study, as our analysis pointed out that heavy smokers with CAD are offered intensive support to stop by SCSs, allowing them to quit despite comorbidities. In fact, SC medications seem not to be prescribed systematically to smokers with MI in routine care, as NRT was prescribed to only 7% of smokers, and varenicline to 2% with nonmeaningful change between the European Action on Secondary and Primary Prevention by Intervention to Reduce Events (EUROASPIRE) III and V studies.^36^^,^^37^

One of the primary strengths of this study is its originality, because few real-life studies have addressed the factors associated with SC in smokers with CAD, according to sex. In addition, systematic validation of self-reported SC was performed by measuring exhaled CO level < 10 ppm at all follow-up consultations.

### Conclusions

Our results support implementation of systematic management of SC in smokers admitted to the cardiology department with MI or angina, as well as referral of these smokers after discharge to an SCS or a rehabilitation department with health professionals trained in SC support to help them maintain their abstinence. Our outcomes support the implementation of tailored interventions that address the specific needs of men and women with CAD who smoke, to help them quit.

## Footnotes

^1^ For this study, sex was considered a binary term—that is, female or male—and individuals who smoked could report their self-representation as being either a woman or a man.
