# Supplementary material for: Factors Associated with Smoking Cessation in Patients with Coronary Artery Diseases According to Sex: Cohort of Smoking Cessation Services Data from France
Source: CJC Open. 2025 Feb 10;7(7):986–96. doi: 10.1016/j.cjco.2025.02.002 (PMC12277821; doi:10.1016/j.cjco.2025.02.002)
Supplement: Supplemental Figure and Tables [file mmc1.pdf]

Supplemental material

Table S1: Outcomes at the end of follow-up according to sex

Characteristics	Women				Men			
	Abstinence, N = 504	Cigarette consumption reduction, N = 224	No change in cigarette consumption, N = 220	p-value	Abstinence, N = 1 932	Cigarette Consumption reduction, N = 872	No change in cigarette consumption, N = 780	p-value
**Age classes, n (%)**								
**Age, mean (sd)**	55 (10)	55 (9)	54 (10)	0.6	55 (9)	56 (9)	54 (9)	<0.001
18-34 years	10 (63)	1 (6.3)	5 (31)		28 (55)	9 (18)	14 (27)	
35-64 years	410 (52)	188 (24)	185 (24)		1 597 (54)	711 (24)	676 (23)	
≥ 65 years	84 (56)	35 (23)	30 (20)		307 (56)	152 (28)	90 (16)	
**Contraceptive Pill, n (%)**	23 (66)	5 (14)	7 (20)	0.3				
**Diploma, n (%)**	345 (56)	147 (24)	121 (20)	0.002	1 445 (55)	666 (25)	520 (20)	<0.001
**Employed, n (%)**	230 (57)	84 (21)	93 (23)	0.12	981 (58)	344 (20)	381 (22)	<0.001
**Personal initiative or encouraged by entourage, n (%)**	137 (56)	59 (24)	47 (19)	0.2	422 (52)	205 (25)	180 (22)	0.6
**Smokes at home, n (%)**	37 (43)	35 (40)	15 (17)	<0.001	94 (43)	81 (37)	43 (20)	<0.001
**Other smoker at home, n (%)**	17 (35)	26 (53)	6 (12)	<0.001	52 (45)	48 (41)	16 (14)	<0.001
**Cardiovascular risk factors, n (%)**								
BMI ≥ 25 kg/m^2	240 (52)	113 (25)	108 (23)	0.8	1 163 (56)	499 (24)	417 (20)	0.005
Arterial hypertension	176 (49)	88 (25)	95 (26)	0.10	635 (52)	321 (26)	272 (22)	0.12
Diabetes	69 (47)	40 (27)	39 (26)	0.2	300 (48)	191 (30)	137 (22)	<0.001
Hypercholesterolemia	211 (54)	92 (24)	87 (22)	0.8	885 (53)	423 (26)	349 (21)	0.3
**Cardiovascular diseases, n (%)**	56 (37)	50 (33)	44 (29)	<0.001	360 (48)	221 (29)	174 (23)	<0.001
**Respiratory diseases, n (%)**	172 (48)	97 (27)	93 (26)	0.023	481 (48)	274 (27)	247 (25)	<0.001
**Psychological disorders, n (%)**								

Depression history	189 (50)	90 (24)	100 (26)	0.13	378 (45)	241 (29)	213 (26)	<0.001
Anxiety and/or depression symptoms[1]	279 (51)	134 (25)	131 (24)	0.4	740 (50)	402 (27)	329 (22)	<0.001
**Psychotropic medications, n (%)**								
Anxiolytics	164 (53)	74 (24)	72 (23)	>0.9	361 (47)	229 (30)	180 (23)	<0.001
Antidepressants	134 (50)	72 (27)	62 (23)	0.3	271 (44)	193 (31)	154 (25)	<0.001
**Smoking-related cancers, n (%)**	24 (53)	11 (24)	10 (22)	>0.9	65 (46)	45 (32)	32 (23)	0.076
**Prior attempt to quit, n (%)**	379 (55)	160 (23)	145 (21)	0.036	1 407 (54)	628 (24)	566 (22)	>0.9
**Cigarettes per day, mean (sd)**	24 (13)	26 (18)	26 (16)	0.6	25 (17)	28 (20)	27 (17)	0.061
**High cigarette consumption[2], n (%)**	207 (52)	94 (24)	94 (24)	>0.9	873 (53)	409 (25)	375 (23)	0.4
**High nicotine dependence[3], n (%)**	323 (53)	145 (24)	145 (24)	0.9	1 128 (52)	543 (25)	496 (23)	0.020
**High confidence in ability to quit[4], n (%)**	179 (58)	71 (23)	60 (19)	0.088	786 (57)	348 (25)	238 (17)	<0.001
**Average CO at the first consultation, mean (sd)**								
**Co-addictions, n (%)**								
Alcohol use disorder	42 (51)	24 (29)	17 (20)	0.5	466 (53)	226 (26)	193 (22)	0.6
Cannabis use	11 (34)	9 (28)	12 (38)	0.067	79 (38)	69 (33)	58 (28)	<0.001
**Opioid substitution treatment, n (%)**	2 (22)	3 (33)	4 (44)	0.12	8 (26)	11 (35)	12 (39)	0.006
**Use of electronic cigarette at the first consultation (dual users), n (%)**	22 (69)	8 (25)	2 (6.3)	0.060	36 (43)	28 (33)	20 (24)	0.083
**Treatment prescribed at the first consultation, n (%)**								
Psychosocial support (PS) No pharmacotherapy	73 (53)	33 (24)	33 (24)		210 (47)	95 (21)	139 (31)	
Transdermal nicotine patches + PS	98 (54)	42 (23)	42 (23)		380 (58)	168 (26)	108 (16)	

[1] Anxiety and/or depression symptoms as measured by the Hospital Anxiety Depression Scale

[2] >20 cigarettes per day
[3] Heaviness of Smoking Index ≥4/6
[4] Visual analog score ≥6/10

Oral nicotine substitutes + PS	44 (34)	37 (29)	48 (37)		194 (40)	150 (31)	137 (28)	
NRT Combination + PS	255 (57)	97 (22)	92 (21)		1 052 (58)	414 (23)	358 (20)	
Varenicline + PS	27 (69)	9 (23)	3 (7.7)		76 (55)	31 (22)	31 (22)	
Varenicline + NRT + PS	5 (38)	6 (46)	2 (15)		16 (46)	14 (40)	5 (14)	
Bupropion + PS	1 (100)	0 (0)	0 (0)		1 (33)	0 (0)	2 (67)	
Bupropion + NRT + PS	1 (100)	0 (0)	0 (0)		3 (100)	0 (0)	0 (0)	
**Number of follow-up consultations**				<0.001				<0.001
mean (sd)	6 (7)	4 (5)	2 (3)	<0.001	5 (6)	4 (5)	2 (2)	<0.001
1 – 3	204 (39)	129 (25)	184 (36)		852 (41)	540 (26)	665 (32)	
4 – 6	153 (64)	58 (24)	28 (12)		562 (68)	191 (23)	75 (9.1)	
≥ 7	147 (77)	37 (19)	8 (4.2)		518 (74)	141 (20)	40 (5.7)	

Table S2: Sex-interaction effect of study variables (Anova test)

	Logistic regression (LR Chisq)	Defines the distribution (Df)	p-value
**sex: Age classes**	0.96	2	0.619075
**sex: Diploma**	0.72	1	0.396783
**sex: Employed**	0.91	1	0.341367
**sex: Personal initiative or encouraged by entourage**	0.04	1	0.833255
**sex: Smoked at home**	0.15	1	0.702053
**sex: Other smoker at home**	3.92	1	0.047811
**sex: BMI ≥ 25 kg/m^2**	3.70	1	0.054524
**sex: Arterial hypertension**	0.36	1	0.549536
**sex: Diabetes**	0.62	1	0.432530
**sex: Hypercholesterolemia**	1.15	1	0.283197
**sex: Other cardiovascular diseases**	4.40	1	0.035921
**sex: Respiratory diseases**	0.61	1	0.433212
**sex: Depression history**	0.02	1	0.892926
**sex: Anxiety and/or depression symptoms**[5]	0.29	1	0.590092
**sex: Anxiolytics**	1.26	1	0.261667
**sex: Antidepressants**	0.55	1	0.459366
**sex: Smoking-related cancers**	0.64	1	0.424639
**sex: Prior attempt to quit**	6.78	1	0.009197
**sex: High cigarette consumption**[6]	0.55	1	0.457357
**sex: High nicotine dependence**[7]	0.30	1	0.583619
**sex: High confidence in ability to quit**[8]	0.02	1	0.902344
**sex: Alcohol use disorder**	0.01	1	0.911726
**sex: Cannabis use**	0.04	1	0.840845
**sex: Opioid substitution treatment**	0.45	1	0.504622
**sex: Use of electronic cigarette at the first consultation**	8.10	1	0.004430
**sex: Treatment prescribed at the first consultation**	13.45	7	0.061937
**sex: Number of follow-up consultations**	0.48	2	0.788546

[5] Anxiety and/or depression symptoms as measured by the Hospital Anxiety Depression Scale
[6] >20 cigarettes per day
[7] Heaviness of Smoking Index ≥4/6
[8] Visual analog score ≥6/10

Table S3: Predictive factors of smoking cessation in women and men, results of the univariate regression model (supplementary material)

Characteristic	Women			Men		
	**OR**	**95% CI**	**p-value**	**ORa**	**95% CI**	**p-value**
**Age**						
18-34 years	—	—		—	—	
35-64 years	0.66	0.22 - 1.79	0.4	0.95	0.54 - 1.65	0.8
≥ 65 years	0.78	0.25 - 2.20	0.6	1.04	0.58 - 1.85	0.9
**Contraceptive Pill**	1.72	0.86 - 3.62	0.13			
**Diploma**	1.42	1.09 - 1.86	0.009	1.17	1.00 - 1.35	0.043
**Employed**	1.27	0.98 - 1.64	0.073	1.32	1.16 - 1.51	<0.001
**Personal initiative or encouraged by entourage**	1.19	0.89 - 1.60	0.2	0.92	0.79 - 1.08	0.3
**Smoked at home**	0.62	0.40 - 0.97	0.038	0.63	0.48 - 0.83	0.001
**Other smoker at home**	0.45	0.24 - 0.81	0.009	0.69	0.47 - 0.99	0.047
**Cardiovascular risk factors**						
BMI ≥ 25	0.92	0.71 - 1.18	0.5	1.22	1.06 - 1.39	0.004
Arterial hypertension	0.77	0.59 - 1.00	0.046	0.87	0.76 - 1.00	0.057
Diabetes	0.73	0.51 - 1.04	0.083	0.74	0.62 - 0.88	<0.001
Hypercholesterolemia	1.07	0.82 - 1.38	0.6	0.96	0.84 - 1.10	0.6
**Cardiovascular diseases**	0.47	0.32 - 0.66	<0.001	0.73	0.62 - 0.86	<0.001
**Respiratory diseases**	0.69	0.53 - 0.90	0.006	0.72	0.62 - 0.83	<0.001
**Psychological disorders**						
Depression history	0.80	0.62 - 1.04	0.10	0.64	0.55 - 0.75	<0.001
Anxiety and/or depression symptoms	0.84	0.65 - 1.08	0.2	0.78	0.68 - 0.89	<0.001
**Psychotropic medications**						
Anxiolytics	0.98	0.75 - 1.29	>0.9	0.70	0.60 - 0.82	<0.001
Antidepressants	0.84	0.63 - 1.11	0.2	0.61	0.52 - 0.73	<0.001
**Smoking-related cancers**	1.01	0.55 - 1.85	>0.9	0.71	0.51 - 1.00	0.048
**Prior attempt to quit**	1.38	1.04 - 1.84	0.026	1.03	0.89 - 1.19	0.7
**High cigarette consumption**	0.95	0.73 - 1.23	0.7	0.91	0.80 - 1.04	0.2
**High nicotine dependence**	0.95	0.73 - 1.24	0.7	0.83	0.72 - 0.95	0.006
**Confidence in ability to quit**	1.32	1.00 - 1.73	0.049	1.25	1.09 - 1.43	0.001
**Co-addictions**						
Alcohol disorder	0.89	0.57 - 1.40	0.6	0.94	0.80 - 1.09	0.4
Cannabis use	0.45	0.21 - 0.92	0.034	0.51	0.38 - 0.68	<0.001
**Opioid substitution treatment**	0.25	0.04 - 1.04	0.083	0.29	0.12 - 0.63	0.003
**Use of electronic cigarette at the first consultation (dual users)**	1.98	0.95 - 4.42	0.077	0.63	0.41 - 0.98	0.041
**Treatment prescribed at the first consultation**						

Psychosocial support (PS) No pharmacotherapy	—	—		—	—	
Transdermal nicotine patches + PS	1.05	0.68 - 1.64	0.8	1.53	1.20 - 1.96	<0.001
Oral nicotine substitutes + PS	0.47	0.28 - 0.76	0.003	0.75	0.58 - 0.98	0.033
NRT Combination + PS	1.22	0.83 - 1.79	0.3	1.52	1.23 - 1.87	<0.001
Varenicline + PS	2.03	0.97 - 4.47	0.066	1.37	0.93 - 2.01	0.11
Varenicline + NRT + PS	0.57	0.16 - 1.78	0.3	0.94	0.46 - 1.87	0.9
Bupropion + PS	704,513	0.00 - NA	>0.9	0.56	0.03 - 5.86	0.6
Bupropion + NRT + PS	704,513	0.00 - NA	>0.9	319,425	0.00 - NA	>0.9
**Number of follow-up visits**						
1 – 3	—	—		—	—	
4 – 6	2.73	1.99 - 3.76	<0.001	2.99	2.52 - 3.55	<0.001
≥ 7	5.01	3.46 - 7.38	<0.001	4.05	3.35 - 4.91	<0.001

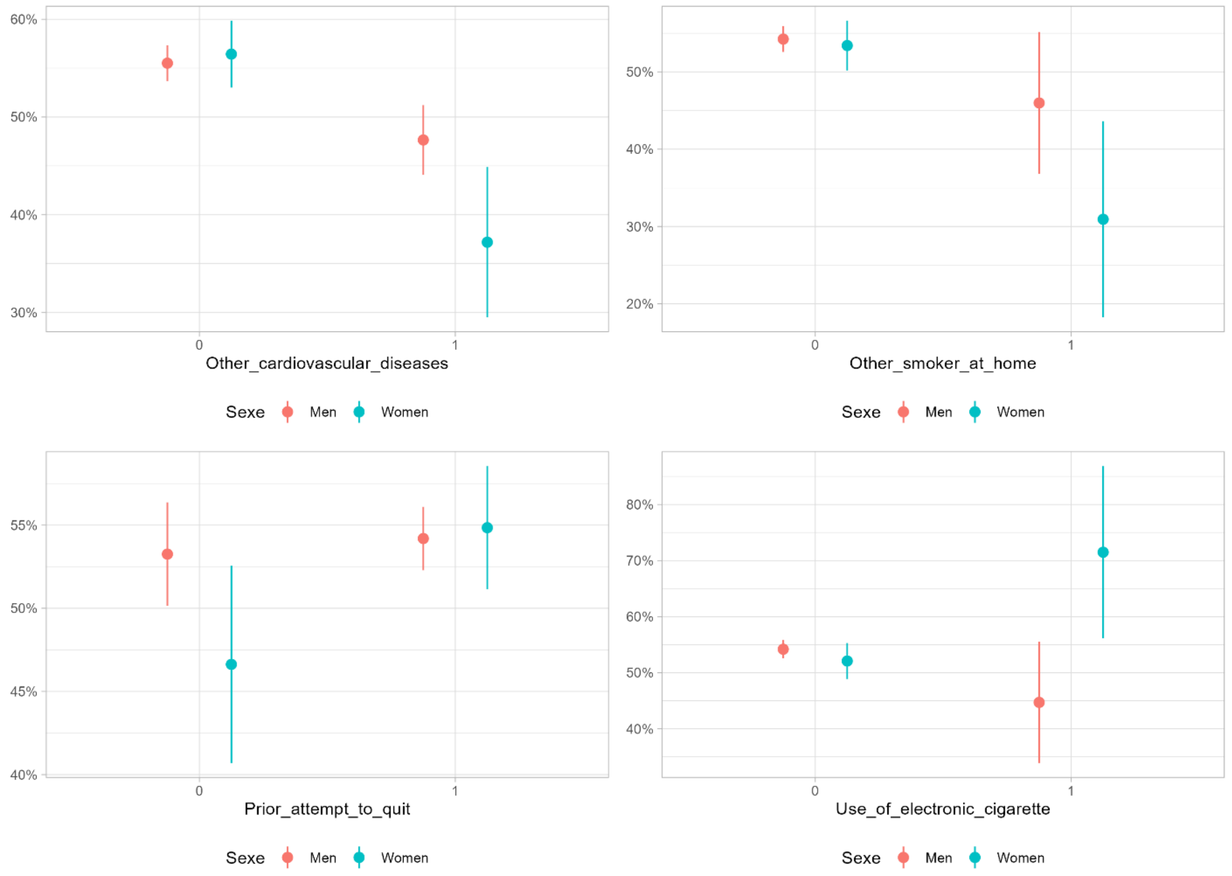

Figure S1: The four variables with sex-interaction
